# Developmental acclimation of the thylakoid proteome to light intensity in *Arabidopsis*

**DOI:** 10.1111/tpj.15053

**Published:** 2020-11-27

**Authors:** Sarah E. Flannery, Christopher Hepworth, William H. J. Wood, Federica Pastorelli, Christopher N. Hunter, Mark J. Dickman, Philip J. Jackson, Matthew P. Johnson

**Affiliations:** ^1^ Department of Molecular Biology and Biotechnology University of Sheffield Firth Court Western Bank Sheffield UK; ^2^ Department of Chemical and Biological Engineering ChELSI Institute University of Sheffield Sheffield UK

**Keywords:** Acclimation, proteomics, thylakoid, light harvesting, electron transfer

## Abstract

Photosynthetic acclimation, the ability to adjust the composition of the thylakoid membrane to optimise the efficiency of electron transfer to the prevailing light conditions, is crucial to plant fitness in the field. While much is known about photosynthetic acclimation in Arabidopsis, to date there has been no study that combines both quantitative label‐free proteomics and photosynthetic analysis by gas exchange, chlorophyll fluorescence and P700 absorption spectroscopy. Using these methods we investigated how the levels of 402 thylakoid proteins, including many regulatory proteins not previously quantified, varied upon long‐term (weeks) acclimation of Arabidopsis to low (LL), moderate (ML) and high (HL) growth light intensity and correlated these with key photosynthetic parameters. We show that changes in the relative abundance of cyt*b*_6_ *f*, ATP synthase, FNR2, TIC62 and PGR6 positively correlate with changes in estimated PSII electron transfer rate and CO_2_ assimilation. Improved photosynthetic capacity in HL grown plants is paralleled by increased cyclic electron transport, which positively correlated with NDH, PGRL1, FNR1, FNR2 and TIC62, although not PGR5 abundance. The photoprotective acclimation strategy was also contrasting, with LL plants favouring slowly reversible non‐photochemical quenching (qI), which positively correlated with LCNP, while HL plants favoured rapidly reversible quenching (qE), which positively correlated with PSBS. The long‐term adjustment of thylakoid membrane grana diameter positively correlated with LHCII levels, while grana stacking negatively correlated with CURT1 and RIQ protein abundance. The data provide insights into how Arabidopsis tunes photosynthetic electron transfer and its regulation during developmental acclimation to light intensity.

## Significance Statement

Developmental acclimation to light intensity in plants involves changes in the organisation and regulation of photosynthetic electron transfer chain components in the chloroplast thylakoid membrane. Here, using mass spectrometry, we quantified changes in the thylakoid proteome and correlated these with key photosynthetic parameters to gain insight into the process.

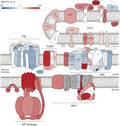

## INTRODUCTION

Plants possess a remarkable ability to react to changes in light intensity, allowing them to flourish in a wide range of environments, from arid sun‐soaked deserts to the deep shade of the rainforest floor (Ruban, 2015). Even within particular environmental niches, the levels of irradiance can fluctuate dramatically according to the season, time of day and meteorological conditions and because of dynamic shading within plant canopies. Changing light intensity affects the balance between solar energy absorption and its utilisation in photosynthesis, potentially leading to metabolic imbalances that trigger photo‐oxidative stress and/or slower growth and development (Foyer and Noctor, 2005; Li *et al*., 2009). Because of this, plants have evolved a complex network of short‐ and long‐term responses to optimise photosynthesis to the prevailing light environment. The short‐term responses take place on a timescale of seconds to minutes and involve regulatory mechanisms that alter the structure and/or function of existing proteins (Tikkanen and Aro, 2014; Ruban, 2016; Theis and Schroda, 2016; Yamori and Shikanai, 2016). In contrast, long‐term acclimation can be categorised as either (i) dynamic acclimation, wherein fully mature leaves undergo *de novo* synthesis and degradation of specific proteins, leading to changes in the organisation of the chloroplast thylakoid membranes, their protein composition and that of the surrounding stroma that contains the enzymes of the CO_2_‐fixing Calvin–Benson cycle (Walters and Horton, 1994; Yin and Johnson, 2000; Athanasiou *et al*., 2010; Suorsa *et al*., 2012), or (ii) developmental acclimation, which is the focus of this study, wherein leaf development and morphology are altered in addition to the changes in chloroplast composition (Boardman, 1977; Anderson, 1986; Anderson *et al*., 1988; Bailey *et al*., 2001; Bailey *et al*., 2004; Walters, 2005; Schöttler and Tóth, 2014; Vialet‐Chabrand *et al*., 2017). The ability to acclimate to light intensity varies not only between species (Murchie and Horton, 1997), but also between different accessions within single species (Athanasiou *et al*., 2010). The signalling pathways that trigger acclimation are not yet fully understood; however, significant roles have been described for the redox state of the electron carrier plastoquinone (Huner *et al*., 1996; Pfannschmidt *et al*., 1999; Rosso *et al*., 2009), the activity of the light‐harvesting complex II (LHCII) kinase STN7 (Pesaresi *et al*., 2009a) and the glucose‐6‐phosphate/phosphate translocator GPT2 (Athanasiou *et al*., 2010). Recent findings demonstrate that acclimation is vital to plant fitness in terms of seed production in fluctuating light environments (Athanasiou *et al*., 2010; Townsend *et al*., 2018).

Developmental acclimation to light intensity in Arabidopsis is known to lead to dramatic changes in the composition of the thylakoid membrane. In low light, plants increase the amount of thylakoid membrane stacking and the ratio of LHCII and photosystem I (PSI) to photosystem II (PSII), whereas high light leads to decreased stacking, reduction in the LHCII/PSII and PSI/PSII reaction centre ratios and increased levels of ATP synthase and cytochrome *b*_6_ *f* (cyt*b*_6_ *f*) complexes relative to total chlorophyll (Walters and Horton, 1995; Bailey *et al*., 2001; Tikkanen *et al*., 2006; Ballottari *et al*., 2007; Wientjes *et al*., 2013a; Wientjes *et al*., 2013b; Ware *et al*., 2015; Schumann *et al*., 2017; Vialet‐Chabrand *et al*., 2017). Consequently, while high light grown plants have a higher overall capacity for linear electron transfer (LET) and CO_2_ assimilation, coupled with an increased resistance to photoinhibition, low light‐acclimated plants utilise low irradiance more effectively (Boardman, 1977; Anderson *et al*., 1988; Gray *et al*., 1996). Until recently, most of the changes in thylakoid composition under different growth light intensities were analysed by either immunoblotting or a range of absorption‐based spectrophotometric assays (Anderson *et al*., 1988; Bailey *et al*., 2001; Schöttler and Tóth, 2014). This body of work has provided valuable insights into the process of acclimation in plants. However, it also suffers from several drawbacks due to limitations of the methods employed. For instance, while qualitatively informative, immunoblotting is subject to many potential pitfalls, has poor quantitative precision and is limited by the availability and specificity of antibodies (Janes, 2015). In addition, while activity measurements can show superior specificity for functional complexes compared to immunoblotting, spectrophotometric assays are in turn limited to components with light‐absorbing cofactors. Moreover, different spectrophotometric assay systems for quantifying the same components often yield substantially different results, hindering direct comparison of data from different studies (Schöttler and Tóth, 2014). Finally, since most of these studies were performed prior to the discovery of numerous key photosynthetic regulatory and structural components, knowledge of how their levels respond to growth light intensity is sparse. In the last decade, developments in high‐throughput high‐resolution mass spectrometry (MS) and the advent of quantitative proteomics, in conjunction with the availability of a well‐annotated *Arabidopsis thaliana* (henceforth Arabidopsis) genome, have facilitated system‐wide analyses of thylakoid protein abundance, which can then be compared to a range of structural and functional data (Aro *et al*., 2004; Friso *et al*., 2004; Peltier *et al*., 2004; Ferro *et al*., 2010; Miller *et al*., 2017). Using this approach a recent study reported the acclimation response of the pea (*Pisum sativum*) thylakoid proteome to three different light intensities and compared this to the functional changes in photosynthesis (Albanese *et al*., 2018). However, since there is no published genome for pea, the number of proteins identified was, by necessity, confined to those sequences that could be retrieved from pre‐existing transcriptomic data. Moreover, the results on pea are not straightforwardly comparable to the large number of functional studies in the literature that use the model organism Arabidopsis. More recently, MS was used to estimate the stoichiometries of the photosynthetic complexes in Arabidopsis for a single moderate light growth condition (McKenzie *et al*., 2020).

Here we have investigated the developmental acclimation of the thylakoid proteome in Arabidopsis using quantitative label‐free proteomic analysis in combination with a range of biochemical and spectroscopic functional analyses. We selected the Arabidopsis Col‐0 ecotype for our acclimation study on the basis of the availability of its well‐annotated proteome database and the large number of photosynthetic mutants that exist in this background. We note that while Col‐0 lacks a significant dynamic acclimation response (Athanasiou *et al*., 2010), it is capable of developmental acclimation (Ballottari *et al*., 2007; Kouřil *et al*., 2013; Wientjes *et al*., 2013b; Ware *et al*., 2015). The use of Arabidopsis Col‐0 facilitated the identification and relative quantification of 402 thylakoid proteins, which we were able to compare to a wider range of structural and functional data to provide insights into developmental acclimation. Furthermore, our approach, involving solubilisation and proteolytic digestion of thylakoids in sodium laurate (SL) detergent (Lin *et al*., 2013), analysis by nano‐flow liquid chromatography coupled to MS (nano‐LC‐MS/MS) and intensity‐based absolute quantification (iBAQ) (Schwanhäusser *et al*., 2011), is simple, straightforward and readily translatable to other photosynthetic organisms with well‐annotated proteome databases.

## RESULTS

### Arabidopsis plants display morphological, biochemical and spectroscopic differences when acclimated to low, medium and high illumination

**Figure 1:**
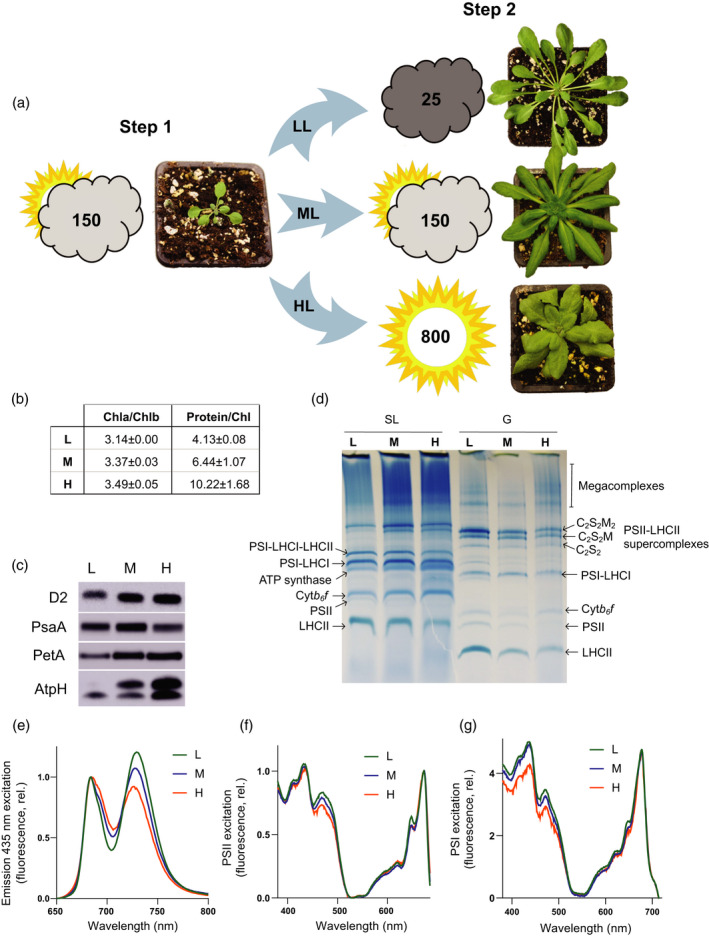
Arabidopsis plants have different morphologies after acclimation under low, moderate and high light. (a) Plants were initially grown under moderate light (ML, Step 1) at 150 µmol photons m^−2^ sec^−1^and then (Step 2) acclimated under low light (LL) at 25 µmol photons m^−2^ sec^−1^or high light at 800 µmol photons m^−2^ sec^−1^(HL) or maintained under ML conditions (see the Experimental Procedures section). The plants were photographed at 2 weeks post‐Step 1, and post‐Step 2 at 7 (LL), 5 (ML) and 4 (HL) weeks. (b) Ratios of Chl *a* to Chl *b* and protein to Chl in isolated thylakoids from LL, ML and HL (*n* = 3, mean ± SD). (c) Immunodetection of D2 (PSII), PSAA (PSI), PETA (cyt *b*_6_ *f*) and ATPH (ATP synthase) in Arabidopsis thylakoid membranes acclimated to LL, ML and HL. Sample loading was normalised to chlorophyll. (d) BN‐PAGE of solubilised stromal lamellae (SL) and granal (G) thylakoid fractions. (e) The 77 K fluorescence emission spectra of LL (green), ML (blue) and HL (orange) thylakoids using 435 nm excitation. (f) The 77 K fluorescence excitation spectra of PSII (695 nm) from LL (green), ML (blue) and HL (orange) thylakoids. (g) The 77 K fluorescence excitation spectra of PSI (735 nm) from LL (green), ML (blue) and HL (orange) thylakoids.

Arabidopsis plants were first grown for 2 weeks at a moderate light intensity (150 μmol photons m^−2^ sec^−1^, ML). Following this 2‐week period, plants were either maintained for a further 3 weeks at ML or alternatively transferred to low light (25 μmol photons m^−2^ sec^−1^, LL) or high light (800 μmol photons m^−2^ sec^−1^, HL) (Figure 1) and thus the leaves expanded and developed under each particular light intensity. The spectrum of the white growth light used is shown in Figure S1. The different growth light intensities had a profound effect on leaf morphology, with LL plants displaying elongated petioles, while HL plants showed truncated petioles and wrinkled leaves compared to ML plants (Figure 1a), as observed previously (Schumann *et al*., 2017). Outwardly, the HL plants displayed no obvious signs of light stress such as accumulation of anthocyanins (Figure 1a). The chlorophyll *a*/*b* ratio of the thylakoids increased with growth light intensity, as did the protein to chlorophyll ratio (Figure 1b). In Figure 1(c), immunoblots against the D2 (PSII), PSAA (PSI), PETA (cyt*b*_6_ *f*) and ATPH (ATP synthase) proteins from an SDS‐PAGE of total thylakoid proteins loaded on an equal chlorophyll basis are shown. Consistent with previous reports, PSII, cyt*b*_6_ *f* and ATP synthase levels increased with growth light intensity relative to total chlorophyll, whereas PSI levels were more constant, with a slight increase seen at ML (Bailey *et al*., 2001; Tikkanen *et al*., 2006). The expected acclimation‐related changes were also clearly observed in the BN‐PAGE analysis at the whole complex level in Figure 1(d). Here, we solubilised thylakoid membranes at equal chlorophyll concentration from LL, ML and HL plants stepwise with digitonin to first remove the unstacked PSI‐enriched stromal lamellae domain of the thylakoids and then solubilised the PSII‐enriched grana that remained with a mixture of *n*‐hexadecyl β‐d‐maltoside and *n*‐dodecyl α‐d‐maltoside (Wood *et al*., 2018). The levels of the ATP synthase complex, recovered in the stromal lamellae fraction, increased with growth light intensity, as did the levels of cyt*b*_6_ *f* (Figure 1d). Changes in the amounts of LHCII were also clearly seen in the BN‐PAGE with fewer free L‐type trimers observed with increasing growth light intensity in both grana and stromal lamellae (Figure 1d). Changes in the amounts of the PSII‐LHCII supercomplexes and their sizes were also observed with growth light intensity, consistent with previous results (Ballottari *et al*., 2007; Kouřil *et al*., 2013; Albanese *et al*., 2016). Within the grana fraction levels of the larger C_2_S_2_M_2_ type PSII‐LHCII supercomplexes, composed of a dimeric PSII RC linked to two copies each of the minor monomeric antenna complexes CP29 (LHCB4), CP26 (LHCB5) and CP24 (LHCB6), and four LHCII trimers, decreased with growth irradiance (Figure 1d). Levels of the smaller C_2_S_2_M‐type supercomplex (lacking one CP24 and one M‐type trimer) and C_2_S_2_‐type supercomplex (lacking both CP24 and both M‐type trimers) were more constant (Figure 1d). We also found that the fraction of C_2_S_2_M_2_ and C_2_S_2_M supercomplexes recovered from the stromal lamellae actually increased in ML and HL compared to LL, suggesting that some redistribution of components between domains occurs with acclimation, or that they are more easily liberated from the grana by digitonin solubilisation (Figure 1d). In the stromal lamellae, the amount of PSI‐LHCI‐LHCII supercomplexes declined with increasing growth light intensity (Figure 1d). The increased antenna cross‐section of PSI in LL is consistent with the larger ratio of the PSI to PSII emission bands observed in the low temperature (77 K) emission spectrum (Figure 1e). The 77 K PSII and PSI excitation spectra (Figure 1f,g) also showed that the antenna cross‐section of each photosystem decreased with increasing growth irradiance.

### Relative quantification of the major photosynthetic complexes by mass spectrometry

**Figure 2:**
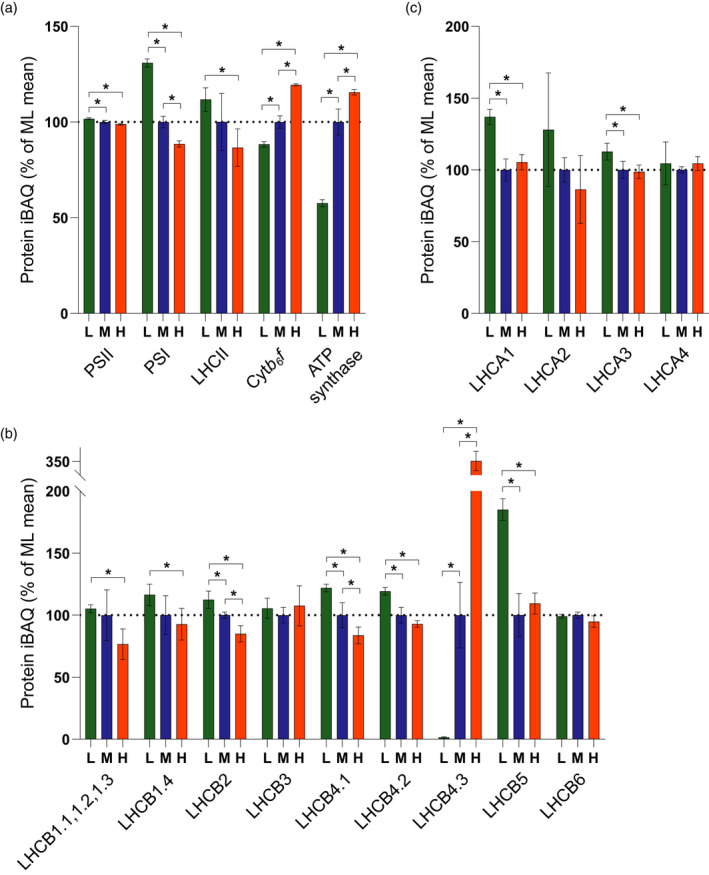
Relative quantification of the major photosynthetic complexes by mass spectrometry. (a) MS analysis showing the relative abundance in low (L), moderate (M) and high (H) light‐acclimated thylakoids of key photosynthetic complexes PSII, PSI, LHCII, cyt *b*_6_ *f* and ATP synthase, expressed as a percentage of the mean at ML. The bars represent the average of three independent peptide preparations (*n* = 3), derived from a pooled thylakoid sample from 15 plants, which were subject to MS analysis in triplicate in a randomised order and the values were averaged. Data are presented as mean ± SD. Significant differences between light conditions were determined by a modified Welch *t*‐test (* *q* < 0.05). (b) MS analysis showing the relative abundance of LHCII trimer and minor monomeric PSII antenna subunits. Sampling details are as stated above. (c) MS analysis showing the abundance of LHCI subunits relative to PSI. Sampling details are as stated above.

Three sets of thylakoid proteins from the LL, ML and HL plants were prepared for proteomic analysis by solubilisation in 1% SL and proteolytic digestion with a combination of endoproteinase Lys‐C and trypsin. The resulting peptide fragments were desalted and analysed by nano‐LC‐MS/MS with data‐dependent acquisition in triplicate. Mass spectra were searched against the UniProtKB Arabidopsis proteome database to identify and quantify a total of 1082 proteins present across all light conditions, of which 402 were positively identified as being thylakoid‐associated. In order to utilise peptide ion intensities as a proxy for protein molar amounts so that multi‐subunit complexes may be relatively quantified, processing methods that compensate for either molecular mass or the number of detectable peptides generated by proteolysis—such as ‘intensity‐based absolute quantification’ (iBAQ) (Schwanhäusser *et al*., 2011)—are required (for a survey of methods, see Fabre *et al*., 2014). Using this approach, we first normalised each dataset to the intra‐analysis sum of the total subunit iBAQ values from proteins of the core photosynthetic machinery: PSI, PSII, ATP synthase and cyt*b*_6_ *f*. Using Perseus software (Tyanova *et al*., 2016), normalised iBAQ values for the three technical repeats were averaged and protein abundances that were affected by light intensity at *q* < 0.05 were identified by a modified one‐way anova. Significant proteins were subjected to a modified Welch *t*‐test (*q* < 0.05) to identify pairs of significant differences for relative quantification of proteins between light conditions. For relative quantification of multi‐subunit protein complexes, the sum of iBAQ intensities from all identified subunits of that complex was used. Relative changes in the intensities of the protein subunits observed are given by complex or protein class in Tables S1 and S2 and are discussed further below. The normalised abundances of the major photosynthetic complexes are presented in Figure 2(a) and displayed relative to ML at 100% for clarity. When normalised to the core photosynthetic proteins the level of PSII is only marginally different between LL, ML and HL in contrast to the data in Figure 1(c,d), which are normalised on a chlorophyll basis. Similarly, while on a chlorophyll basis PSI abundance does not correlate with light intensity (Figure 1c,d), on a protein basis it increases by approximately 30% in LL and decreases by approximately 15% in HL (Figure 2a). LHCII increases in LL by approximately 10% and decreases by approximately 15% in HL, cyt*b*_6_ *f* decreases by 15% in LL and increases by 20% in HL and ATP synthase decreases by 45% in LL and increases by approximately 20% in HL (Figure 2a).

The change in the relative abundance of the major trimer LHCII subunits LHCB1, 2 and 3 and minor monomeric antenna subunits 4, 5 and 6 determined by MS is presented in Figure 2(b). Of the five LHCB1 isoforms (LHCB1.1–1.5) in the Arabidopsis genome (Pietrzykowska *et al*., 2014) LHCB1.1, 1.2 and 1.3 did not produce unique peptides to allow them to be individually distinguished and the relative abundance in Figure 2(b) is therefore representative of their collective level, which decreased approximately 25% in HL but was unchanged in LL compared to ML. LHCB1.4 did produce unique peptides and so could be separately quantified, increasing by approximately 10% in LL relative to ML and HL (Figure 2b), whereas LHCB1.5 was not identified in our MS analysis. The sequence similarities of the LHCB2.1, 2.2 and 2.4 isoforms prevent differentiation but collectively they increased by 10% in LL and decreased by 20% in HL (Figure 2b). LHCB3, the less abundant LHCII trimer constituent found mainly in the M‐type trimer (Caffarri *et al*., 2009) remained constant in each light condition (Figure 2b). Similarly, the levels of the minor monomeric antenna complex LHCB6 that is most closely associated with the M‐trimer were also relatively constant across the three light intensities (Figure 2b). These results contrast with studies on Arabidopsis (Ballottari *et al*., 2007; Kouřil *et al*., 2013) which showed LHCB3 and 6 both decrease on a chlorophyll basis under high growth light (Ballottari *et al*., 2007), but are consistent with another study which showed they remained relatively unchanged (Bailey *et al*., 2001) (Figure 2b). The levels of LHCB5 increased markedly in LL, a result previously seen in Arabidopsis (Bailey *et al*., 2001). The content of LHCB4.1 and 4.2 showed a 15% and 5% decrease in HL, respectively, and both showed a 15% increase in LL (Figure 2b). In contrast, the minor LHCB4.3 isoform increased dramatically (+255%) in HL, consistent with observations of Miller *et al*.(2017) and Albanese *et al*.(2018), and decreased by 95% in LL (Figure 2b).

The change in the relative abundance of the LHCI subunits LHCA1, 2, 3 and 4 is presented in Figure 2(c). The high‐resolution PSI‐LHCI complexes from maize (*Zea mays*) (Pan *et al*., 2018) and pea (Mazor *et al*., 2015; Qin *et al*., 2015) show a stoichiometry of 1:1:1:1 for PSI relative to LHCA1, 2, 3 and 4. Unlike PSII, where changes in antenna size have been consistently observed, the LHCI antenna size of PSI has been reported to be unaffected by changes in light intensity in some studies (Ballottari *et al*., 2007; Albanese *et al*., 2018), but altered in another (Bailey *et al*., 2001). Indeed, a recent study showed that PSI in Arabidopsis can bind additional copies of LHCA1 and 4 (Crepin *et al*., 2020), suggesting the antenna size can undergo acclimation. Consistent with this suggestion, we observed a 10–20% increase in LHCA1 and 3 in LL compared to ML but no significant change in HL (Figure 2c). In our MS analysis the minor LHCA5 and 6 proteins, which are involved in binding the NDH complex to PSI (Peng *et al*., 2009; Yadav *et al*., 2017), were not detected.

### Thylakoid membrane grana diameter is positively correlated with LHCII abundance, grana stacking is negatively correlated with CURT1A, B and RIQ1, 2 abundance

**Figure 3:**
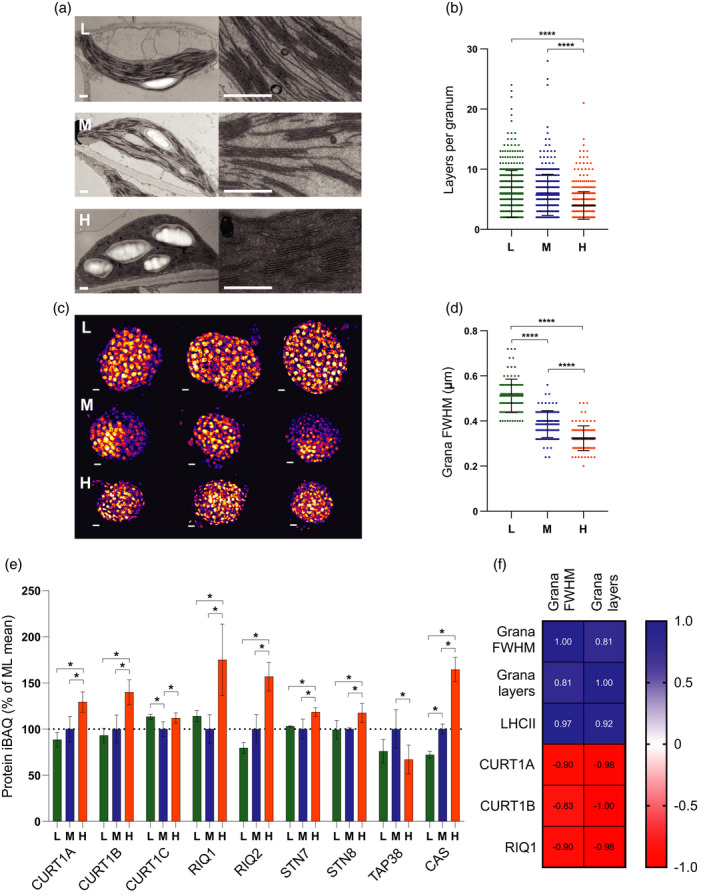
Assessment of changes in thylakoid membrane grana size during developmental acclimation. (a) Thin section electron micrographs of chloroplasts in plants acclimated to LL (top row, L), ML (middle row, M) and HL (bottom row, H) (scale bar: 0.5 µm). (b) Number of membrane layers per grana stack calculated from electron microscopy images of chloroplasts in LL (*n* = 379 grana stacks), ML (*n* = 354) and HL (*n* = 507) leaves (one‐way anova with Tukey’s multiple comparisons, ****P < 0.0001). Data are presented as mean ± SD. (c) 3D‐SIM images (shown as Max Projections on the *z*‐axis with tricubic sharp interpolation) of chloroplasts in plants acclimated to LL (top row, L), ML (middle row, M) and HL (bottom row, H) (scale bar: 0.5 µm). (d) Full‐width at half‐maximum (FWHM) fluorescence intensity of the fluorescent spots (grana) in three‐dimensional SIM images of chloroplasts in LL (*n* = 97), ML (*n* = 100) and HL (*n* = 100) leaves (one‐way anova with Tukey’s multiple comparisons, ****P < 0.0001). Data are presented as mean ± SD. (e) MS analysis showing the relative abundance of proteins involved in the modulation of thylakoid membrane architecture, expressed as a percentage of the mean at ML. Sampling details are as stated in Figure 2. (f) Pearson correlation of the mean number of membrane layers per granum and grana FWHM with protein iBAQ values of LHCII trimers, CURT1A, CURT1B and RIQ1. Blue panels indicate a positive correlation while red panels indicate a negative correlation.

Using thin‐section electron microscopy (EM) we observed increased grana thylakoid stacking (membrane layers per granum) in plants grown in LL compared to ML, while HL plants showed a significant decrease (Figure 3a,b), consistent with observations in a number of different plant species (Chow and Anderson, 1987; Chow and Hope, 1987; Chow *et al*., 1988; Bailey *et al*., 2001; Ballottari *et al*., 2007; Petersen *et al*., 2011; Miller *et al*., 2017; Schumann *et al*., 2017). More recently, changes in grana stacking have been found to be accompanied by changes in grana diameter (Pietrzykowska *et al*., 2014; Wood *et al*., 2019, 2018). Consistent with these changes, analysis of chloroplast ultrastructure by structured illumination microscopy (SIM) revealed that increased grana stacking in LL leaves was paralleled by an increase in the grana diameter (measured as the full‐width half‐maximum [FWHM] of the chlorophyll fluorescence signal from each granum) (Figure 3c,d). Similarly, decreased grana stacking in HL leaves was accompanied by a reduction in grana diameter (Figure 3c,d). Previously, changes in the degree of grana stacking have largely been attributed to alterations in the content of LHCII proteins, since interactions between their stromal faces are known to mediate this phenomenon (Day *et al*., 1984). However, more recently the thylakoid curvature protein family (CURT1) was shown to exert a major influence on thylakoid structure, with the *curt1abcd* mutant showing grossly enlarged pseudo‐grana, despite similar levels of LHCII (Armbruster *et al*., 2013). In contrast, Arabidopsis plants overexpressing CURT1A showed smaller grana than the wild‐type (Armbruster *et al*., 2013). Our MS analysis allowed us to quantify how the levels of these key proteins changed upon light acclimation (Figure 3e). The relative abundance of CURT1A and B increased by 30% and 40%, respectively, in HL compared to ML, while in LL both decreased by approximately 10%. There were small increases of approximately 10% in the level of CURT1C in both LL and HL relative to ML (Figure 3e) and CURT1D was not detected. The reduced induction of quenching (RIQ) proteins RIQ1 and 2 have been shown to negatively regulate grana size (Yokoyama *et al*., 2016). Consistent with this earlier finding, the relative abundance of RIQ1 and 2 increased by 75% and 50%, respectively, in HL compared to ML, while RIQ2 decreased by approximately 20% in LL (Figure 3e). A correlation of grana size with the MS data (Figure 3f) showed that the amount of LHCII was most strongly correlated (positively) with grana diameter, whereas the CURT1 and RIQ proteins were most strongly correlated (negatively) with the number of layers per granum. There is evidence that phosphorylation of PSII and LHCII also strongly influences grana stacking, with mutants lacking the PSII (STN8) and LHCII (STN7) kinases showing larger grana, while those lacking the LHCII (TAP38) phosphatase show smaller grana (Fristedt *et al*., 2009; Armbruster *et al*., 2013; Wood *et al*., 2019). Our MS analysis also revealed that the relative abundances of STN7 and STN8 were similar in ML and LL but both increased slightly in HL acclimated plants. In contrast, the relative abundance of TAP38 decreased to a similar extent in both LL and HL plants compared to ML (Figure 3e). The calcium sensor kinase protein CAS, which also plays a role in regulating photo‐acclimation in high light by promoting dephosphorylation of LHCII (Cutolo *et al*., 2019), also increased in abundance in HL by approximately 70% and decreased in LL by approximately 30% (Figure 3e).

### Growth irradiance‐dependent increases in linear electron transfer capacity and CO_2_ assimilation are positively correlated with increased abundance of cyt*b*_6_ *f*, PGR6, FNR2, TIC62 and ATP synthase

**Figure 4:**
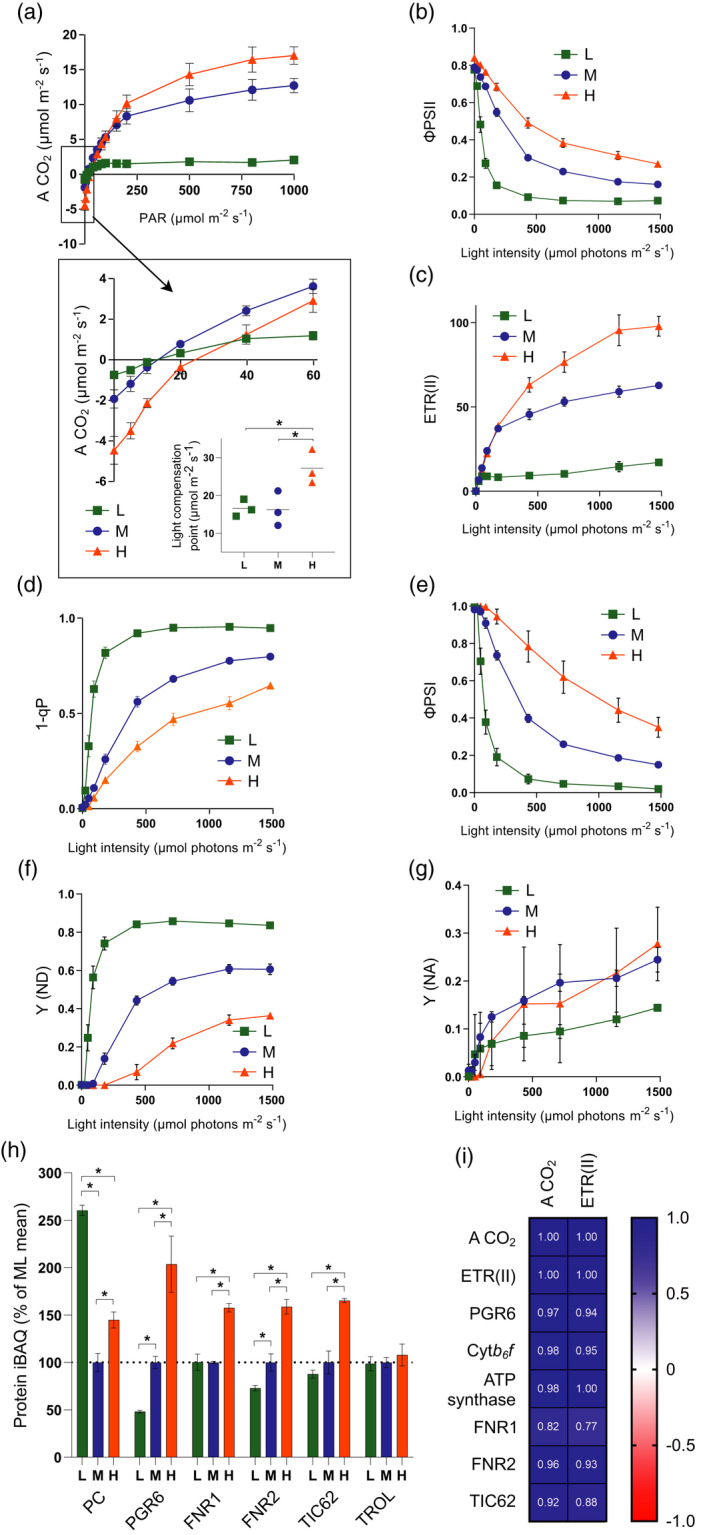
Assessment of changes in linear electron transfer (LET) capacity and CO_2_ assimilation during developmental acclimation. (a) A CO_2_ as a function of light intensity. Inset highlights gas exchange under low light and the calculated light compensation point. (b) PSII light use efficiency (ΦPSII) as a function of light intensity. (c) Estimated electron transfer rate of PSII (ETR(II)) as a function of light intensity. (d) The fraction of closed PSII reaction centres (1 − qP). (e) PSI light use efficiency (ΦPSI) as a function of light intensity. (f) PSI donor side limitation (Y (ND)). (g) PSI donor side limitation (Y (NA)). (h) MS analysis showing the relative abundance of electron transfer proteins, expressed as a percentage of the mean at ML. Sampling details are as stated in Figure 2. (i) Pearson correlation of the maximum A CO_2_ and ETR(II) with protein iBAQ values of PGR6, cyt*b*_6_ *f*, ATP synthase, FNR1, FNR2 and TIC62. Blue panels indicate a positive correlation while red panels indicate a negative correlation. For (a–g), HL = orange, ML = blue, LL = green. *n* = 4, with the exception of (a), where *n* = 3. Asterisks denote significance (one‐way anova with Tukey’s multiple comparisons, *P < 0.05). Error bars denote SEM.

Using chlorophyll fluorescence, P700 absorption spectroscopy and infra‐red gas exchange analysis, we recorded light response curves for the LL, ML and HL plants to assess their light utilisation efficiency, as reflected in the major photosynthetic parameters. It is important to point out that the actinic light used to obtain these curves was a mixture of 10% 460 nm and 90% 635 nm light rather than the broader spectrum fluorescent white lights the plants were grown under (Figure S1), and therefore the light intensities are not directly comparable. The CO_2_ assimilation rate (A CO_2_) was highest in the HL plants, followed by ML and LL, demonstrating successful acclimation (Figure 4a). Consistent with this, PSII light use efficiency (ΦPSII) was increased in HL plants at all but the lowest light intensity measured (Figure 4b) and in line with this the estimated PSII electron transfer rate (ETR(II)) was approximately 5 times higher in HL plants at 1479 μmol photons m^−2^ sec^−1^ compared to LL plants and approximately 2 times higher than ML plants (Figure 4c). It was notable that LL plants showed increased A CO_2_ at the three lowest light intensities (4, 9 and 14 μmol photons m^−2^ sec^−1^) compared to ML and HL (Figure 4a, inset). Since there was no significant difference in ETR(II) under low light intensities (Figure 4c), the higher A CO_2_ of LL plants under low irradiance can be attributed to a lower rate of respiration, consistent with their lower light compensation point (Figure 4a, inset). The lower ETR(II) in LL plants above 20 μmol photons m^−2^ sec^−1^ is associated with an increased PSII acceptor side limitation (1 − qP) (Figure 4d), indicating less efficient oxidation of Q_A_^−^ compared to HL plants, with ML plants falling in between. The PSI light use efficiency (ΦPSI) was greater across all but the lowest light intensity used (Figure 4e). The lower ΦPSI in LL plants could be explained in part by a greater donor side limitation (Y(ND)) above 20 μmol photons m^−2^ sec^−1^ compared to ML plants, with HL plants least affected (Figure 4f). The PSI acceptor side limitation (Y(NA)) was slightly higher in the HL and ML plants compared to LL, likely reflecting the much stronger Y(ND) in the latter (Figure 4g).

Our MS analysis showed a significant increase in the relative abundance of cyt*b*_6_ *f* and ATP synthase with growth light intensity (Figure 2a) and these complexes were positively correlated with ETR(II) and A CO_2_ (Figure 4i). PGR6, a protein kinase associated with the plastoglobuli that acts to increase the number of photoactive plastoquinone molecules inside the thylakoid (Figure 4h) (Pralon *et al*., 2019), also strongly increased with growth light intensity (Figure 4h) and positively correlated with ETR(II) and A CO_2_ (Figure 4i). Ferredoxin‐NADP^+^ reductase catalyses the final step in LET, transferring electrons from ferredoxin (Fd) to NADP^+^, and its antisense inhibition strongly affected photosynthetic capacity in tobacco (*Nicotiana benthamiana*) (Hajirezaei *et al*., 2002). The two isoforms of FNR in Arabidopsis (FNR1 and FNR2) can exist in two states, either soluble in the stroma or bound to the thylakoid membrane via the TIC62 (Benz *et al*., 2009) or TROL (Jurić *et al*., 2009) tethering proteins. Both FNR1 and 2 in addition to the TIC62 protein showed changes in relative abundance with growth irradiance. In contrast to TIC62, no significant effect of light intensity on levels of the TROL protein was observed (Figure 4h). FNR2 and TIC62 showed a positive correlation with A CO_2_ and ETR(II), whereas this was much weaker for FNR1 (Figure 4i). Interestingly, the relative abundance of the PSI electron donor plastocyanin (PC) was significantly higher in LL than in ML and HL plants (Figure 4h).

### Cyclic electron transfer capacity increases with growth irradiance and is positively correlated with PGRL1A, PGRL1B, NDH, FNR1, FNR2 and TIC62 abundance

**Figure 5:**
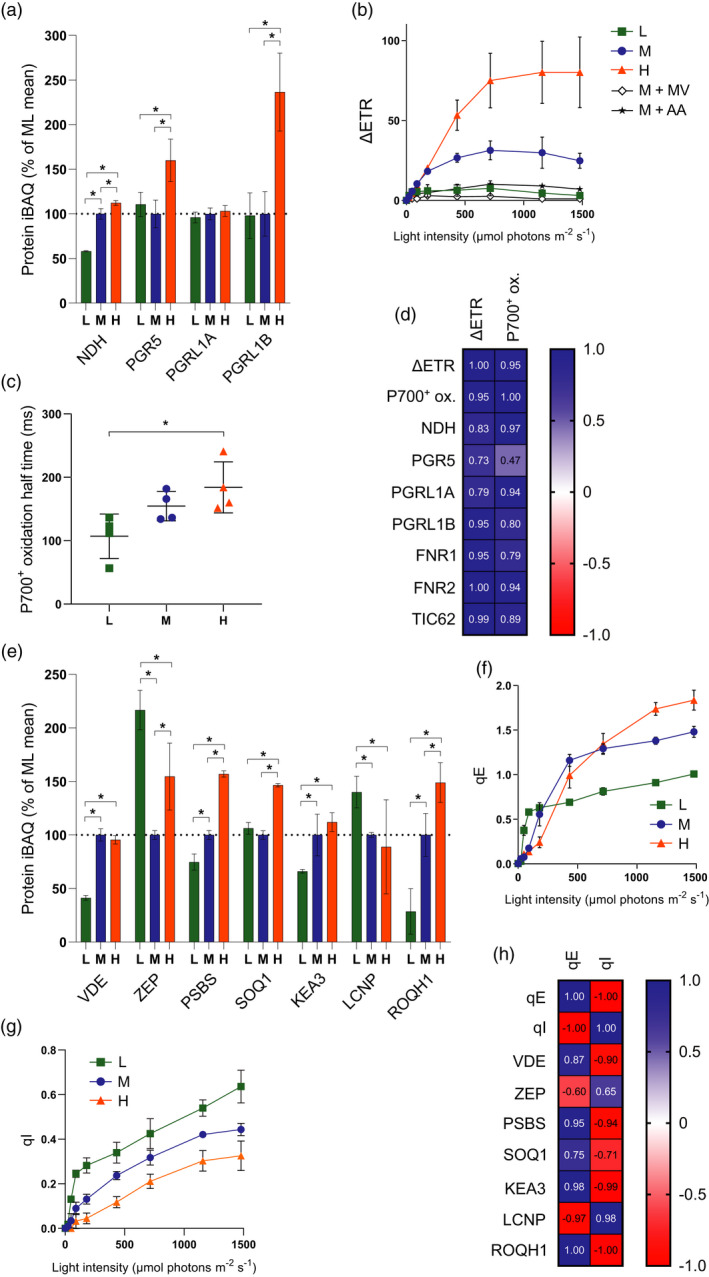
Assessment of changes in cyclic electron transfer (CET) and non‐photochemical quenching (NPQ) during developmental acclimation. (a) MS analysis showing the relative abundance of CET‐related proteins, expressed as a percentage of the mean at ML. Sampling details are as stated in Figure 2. (b) Difference in estimated electron transfer rate (ΔETR) between PSI (ETR(I)) and PSII (ETR(II)) versus light intensity. (c) P700 oxidation half‐time upon illumination with 255 μmol photons m^−2^ sec^−1^ 740 nm light (one‐ way anova with Tukey’s multiple comparisons, *P < 0.05). (d) Pearson correlation of the maximum ΔETR and P700^+^ oxidation half‐time with protein iBAQ values of NDH, PGR5, PGRL1A, PGRL1B, FNR1, FNR2 and TIC62. (e) MS analysis showing the relative abundance of NPQ‐related proteins, expressed as a percentage of the mean at ML. Sampling details are as stated in Figure 2. (f) Rapidly reversible NPQ (qE) of chlorophyll fluorescence versus light intensity. (g) Slowly reversible NPQ (qI) of chlorophyll fluorescence versus light intensity. Sampling details are as stated in Figure 4 and are the same for (b), (c), (f) and (g). For (b), data for ML leaves infiltrated with methyl viologen and with antimycin A are indicated by M + MV and M + AA, respectively. (h) Pearson correlation of the maximum qE and qI with protein iBAQ values of NPQ‐related proteins.

While LET produces ATP and NADPH, which is mostly consumed by the Calvin–Benson cycle in the stroma for CO_2_ fixation, cyclic electron transfer (CET) produces only ATP and as such may play a key role in balancing the ATP/NADPH budget in the chloroplast under different light conditions (Kramer and Evans, 2011). Since CET increases proton flux into the lumen it may also be important for the down‐regulation of PSII and PSI activity by non‐photochemical quenching (NPQ) and photosynthetic control, respectively (Ruban, 2016; Theis and Schroda, 2016; Yamori and Shikanai, 2016). Two pathways of CET, which involve the recycling of electrons from Fd at the PSI acceptor side to the PQ pool, are thought to exist. The first involves the NADPH dehydrogenase‐like complex (NDH), a multi‐subunit proton‐pumping Fd‐PQ oxidoreductase (FQR), while the second involves proton gradient regulation complex proteins PGR5 and PGRL1, which may act directly as an FQR or regulate putative CET activity of a Fd‐FNR‐cyt*b*_6_ *f* complex (Joliot and Johnson, 2011; Hertle *et al*., 2013; Yamori and Shikanai, 2016; Buchert *et al*., 2020). Our MS analysis showed contrasting light acclimation responses for these CET proteins (Figure 5a). The relative abundance of NDH complex decreased by 50% in LL and increased by 10% in HL relative to ML (Figure 5a). In contrast, the relative abundance of PGR5 was similar in LL and ML but increased by approximately 50% in HL (Figure 5a). Despite their high sequence similarity, the relative abundance of PGRL1B increased by 150% in HL whereas PGRL1A showed no significant increase (Figure 5a). Neither protein decreased in LL relative to ML (Figure 5a). Comparison of ETR(I) with ETR(II) allows an estimate of the amount of excess PSI turnover (ΔETR = (ETR(I) − ETR(II))) to be made at each light intensity, which may reflect the contributions of CET (Kou *et al*., 2013) and/or charge recombination in PSI (Kadota *et al*., 2019) (Figure 5b). We performed two controls to assess whether ΔETR is a suitable proxy for CET under our conditions, infiltrating ML leaves with either methyl viologen (MV) or antimycin A (AA), two inhibitors of CET. In MV infiltrated leaves ΔETR was reduced to less than 10% of the ML control, while in AA infiltrated leaves the level was approximately 30%, the remaining ΔETR possibly reflecting the AA‐insensitive NDH‐dependent CET pathway. Comparing plants grown at different irradiance, ΔETR increases in HL plants as a function of light intensity and the maximum amplitude is much larger in these plants, whereas initial increases in LL and ML plants are followed by slight decreases at higher intensity (Figure 5b), which may reflect the well‐known necessity for ‘redox poise’, with over‐reduction of the PQ pool inhibiting CET (Yamori and Shikanai, 2016). We also assessed the capacity for CET by measuring the rate of P700 oxidation with far‐red light (740 nm) that preferentially excites PSI (Joliot and Johnson, 2011) (Figure 5c). In dark‐adapted leaves where the Calvin–Benson cycle is inactive, the delay in far‐red induced P700 oxidation is thought to reflect the efficient re‐reduction of P700^+^ by cycled electrons (Joliot and Johnson, 2011). Consistent with the increased CET capacity inferred by ΔETR in HL plants, the oxidation half‐time of PSI was longest in the HL plants, followed by the ML and LL grown plants (Figure 5c; Figure S2). Interestingly, NDH shows much stronger correlation with CET capacity—particularly when measured by P700 oxidation—than PGR5 (Figure 5d). In contrast PGRL1A, FNR1 and 2 and TIC62 showed the strongest positive correlation with the ΔETR method of assessing CET (Figure 5d).

### Rapidly reversible non‐photochemical quenching (qE) is positively correlated with PSBS and KEA3 abundance, slowly reversible non‐photochemical quenching (qI) is positively correlated with LCNP and negatively correlated with ROQH1 abundance

As light intensity rises, the higher electron transfer activity results in an increased movement of protons from the stroma to the lumen. The influx of protons via coupled LET and CET activity is balanced by proton efflux via the ATP synthase and H^+^/K^+^ antiporter KEA3 (Armbruster *et al*., 2014). Where proton influx exceeds efflux, the increasing ΔpH triggers the activation of NPQ, a mechanism of photoprotective energy dissipation in the PSII antenna system that reduces the excitation pressure on PSII reaction centres. NPQ is comprised of several components (Ruban, 2016), the major two being the rapidly reversible qE form and the slowly reversible qI form. The qE form is triggered by the protonation of both violaxanthin de‐epoxidase (VDE), which converts the LHCII‐bound xanthophyll violaxanthin to zeaxanthin, and the PSBS protein (Ruban, 2016). Together PSBS and zeaxanthin induce conformational changes in LHCII that result in the formation of dissipative chlorophyll–carotenoid or chlorophyll–chlorophyll interactions, protecting PSII from photo‐oxidative damage (Li *et al*., 2009; Ruban, 2016). HL plants showed increased levels of qE compared to ML and LL plants (Figure 5f), although qE developed at lower light intensity in the LL grown plants. Our MS analysis also showed that abundance of PSBS increased markedly with light intensity (Figure 5e) and showed a positive correlation with qE (Figure 5h). In contrast, VDE was constant between ML and HL but decreased in LL and was poorly correlated with qE (Figure 5e,h). The abundance of zeaxanthin epoxidase (ZEP), which catalyses the reconversion of zeaxanthin to violaxanthin, increased in both LL and HL (Figure 5e). The K^+^/H^+^ antiporter protein KEA3, which modulates the relaxation of ΔpH upon high to low light transitions (Armbruster *et al*., 2014), also positively correlated with qE (Figure 5e,h). In contrast with the increased capacity of qE in HL and ML plants, the LL plants showed a higher level of qI at all light intensities. The qI component is complex, involving contributions from both photoinhibition, sustained zeaxanthin‐dependent quenching and the recently discovered LCNP‐dependent quenching (Ruban *et al*., 2012; Malnoë *et al*., 2018). LCNP is a luminal located lipocalin protein, involved in promoting the sustained slowly relaxing component of qI termed qH (Malnoë *et al*., 2018). Interestingly, LCNP showed the highest abundance in LL and moreover was in good correlation with qI (Figure 5h), while SOQ1 and ROQH1, which suppress qH (Brooks *et al*., 2013; Amstutz *et al*., 2020), were more abundant in HL, the latter negatively correlating with qI. Therefore, the HL grown plants appear to reduce their capacity for the slowly relaxing qH form of NPQ, while increasing their capacity for the rapidly relaxing qE form compared to LL (Figure 5f).

### Acclimation to high light leads to increased abundance of the PSII repair cycle machinery

**Figure 6:**
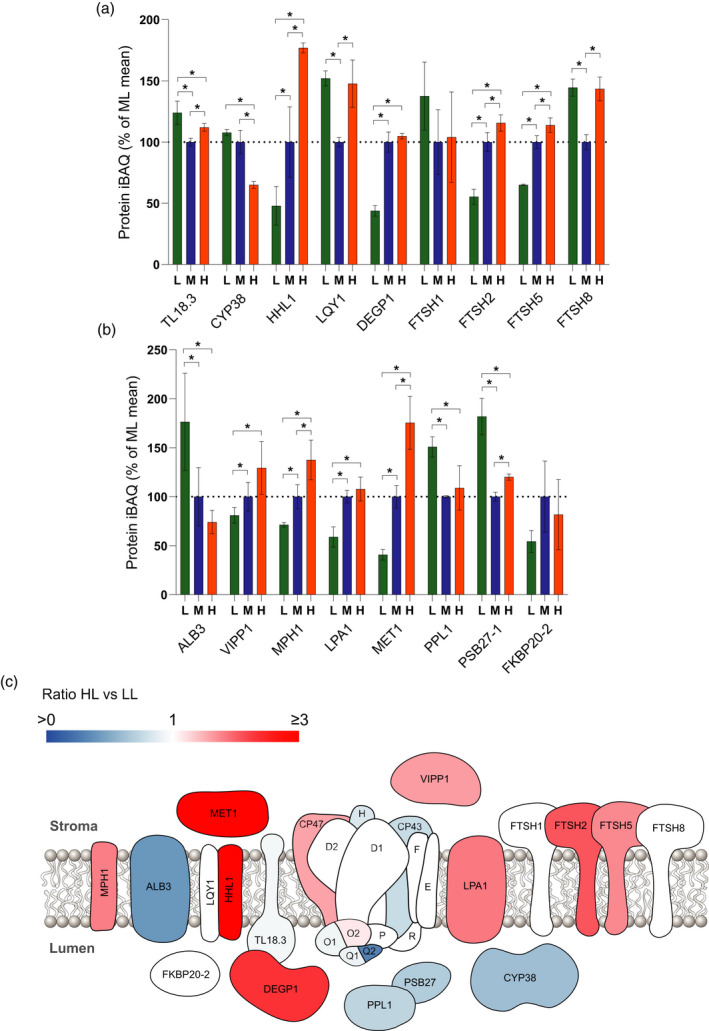
Changing abundance of the PSII repair cycle machinery upon developmental acclimation. (a,b) MS analysis showing the relative abundance of proteins involved in PSII repair, expressed as a percentage of the mean at ML. Sampling details are as stated in Figure 2. (c) Diagram indicating the abundance of PSII repair proteins in HL versus LL. Blue proteins are more abundant in LL, whereas red/pink proteins are more abundant in HL. For quantified proteins where no significant difference was detected, they are displayed in white.

In high light, PSII is prone to photo‐oxidative damage, particularly to the reaction centre D1 subunit (reviewed in Theis and Schroda, 2016). Photodamaged PSII is repaired via a complex repair cycle involving the migration of PSII from the grana to stromal lamellae, partial disassembly of the PSII core and associated OEC, proteolytic excision of D1, *de novo* synthesis of D1, its reinsertion into the PSII complex, and the subsequent reassembly of the dimeric PSII before it is returned to the grana (Aro *et al*., 1993). Using MS, we determined the effect of growth light intensity on the relative abundance of proteins involved in the repair cycle (Figure 6a,b). Phosphorylation of the PSII core proteins D1, D2, PSBH and CP43 by STN8 is thought to promote the migration of photodamaged PSII to the stromal lamellae for repair (Tikkanen *et al*., 2008) and its relative abundance was increased in HL plants (Figure 3e). Prior to repair, PSII must be dephosphorylated by PBCP and possibly TL18.3; the former was not detected in our study but the relative abundance of the latter was fairly constant between LL and HL (Figure 6a). This process may be instead enhanced via decreases in the level of the immunophilin CYP38, which negatively regulates PSII core phosphatase activity (Vener *et al*., 1999), and indeed its relative abundance significantly decreased in HL compared to LL (Figure 6a). The abundance of HHL1 and LQY1, which mediate the release of CP43 from photodamaged PSII prior to D1 proteolysis (Jin *et al*., 2014), behaved differently; the former was significantly increased in ML and HL compared to LL, while the relative abundance of the latter was increased in both LL and HL compared to ML (Figure 6a). Consistent with a greater role for the repair cycle, the relative abundance of the DEGP1 protease was markedly increased in ML and HL (Figure 6a). The behaviour of the FTSH zinc metalloproteinase subunits was more complex; FTSH2 and FTSH5 increased as expected in ML and HL, but there was no observed change in FTSH1 (Figure 6a). FTSH8 showed the same behaviour as LQY1 increasing under both LL and HL compared to ML. The content of the membrane insertase ALB3 (Schneider *et al*., 2014) significantly decreased in ML and HL compared to LL, while VIPP1, which is involved in the formation of lipidic microdomains to assist insertase activity, increased with growth light intensity (Liu *et al*., 2005) (Figure 6b). The MPH1 protein has been implicated in the protection of PSII from photodamage rather than in PSII repair (Theis and Schroda, 2016) and accordingly its relative abundance increased in ML and HL. The LPA1 and MET1 proteins, which function as chaperones in PSII assembly (Theis and Schroda, 2016), also showed significant increases with growth light intensity. Lumenal protein PPL1, which has an as‐yet undefined role in the PSII repair cycle, decreased in HL and ML relative to LL (Figure 6b), despite the fact that Arabidopsis mutants lacking this protein show slower PSII recovery following excess illumination (Ishihara *et al*., 2007). Proteins involved in the reassembly of the Mn cluster, such as PSB27, were lowest in ML, while there was no significant change in FKBP20‐2, which is involved in reassembly of PSII supercomplexes (Theis and Schroda, 2016). The collective changes of the PSII repair cycle machinery proteins in HL relative to LL are illustrated by the schematic diagram in Figure 6(c).

## DISCUSSION

**Figure 7:**
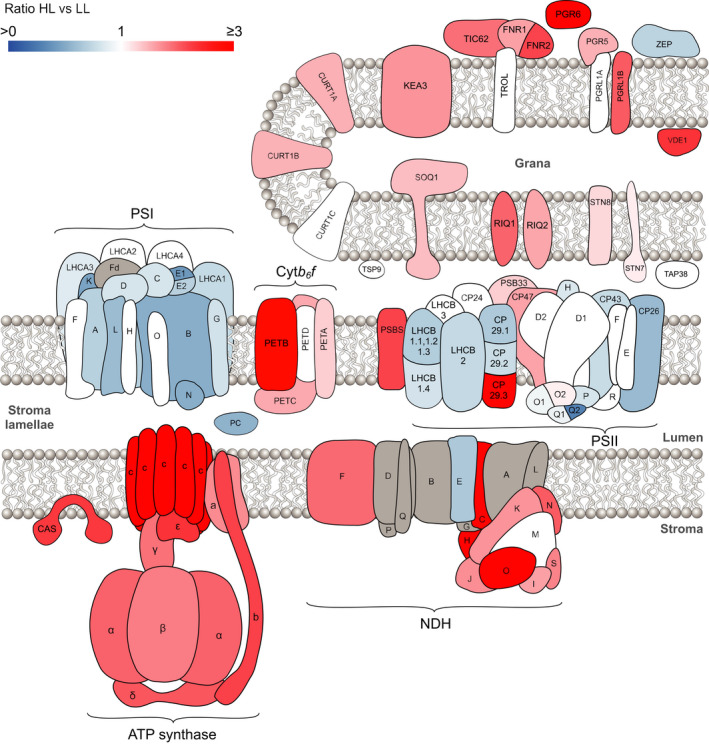
A comparison of high light versus low light acclimation in the thylakoid membrane proteome. Schematic diagram indicating the relative abundance of thylakoid proteins in HL versus LL. Blue proteins are more abundant in LL, whereas red/pink proteins are more abundant in HL. Where no significant difference was detected for a quantified protein it is displayed in white. Proteins not identified by MS analysis are shown in grey.

In this study, we have combined quantitative MS with functional and structural analyses to provide one of the most in‐depth views of developmental acclimation in Arabidopsis yet reported. No previous study of plant acclimation has quantified changes in relative abundance of such a large number (402) of thylakoid proteins and then correlated these with key photosynthetic parameters. Given the demonstrable importance of acclimation to plant fitness, this work represents a valuable resource for the community that can inform future efforts to manipulate this process for crop improvement (Athanasiou *et al*., 2010; Townsend *et al*., 2018). The iBAQ method of quantification that we utilised accounts for how differences in protein size can affect MS intensities and is unaffected by variable sample complexity. This means that iBAQ data can be used for normalisation by division, either to the whole proteome or to a majority subset of the proteins quantified in an experiment. We utilised this approach, normalising each dataset to the intra‐analysis sum of iBAQ intensities for the combination of PSI, PSII, cyt*b*_6_ *f* and ATP synthase, which represent 50–60% of total protein according to iBAQ. The data in this paper thus offer an interesting counterpoint to previous acclimation studies that generally normalised protein abundance on a chlorophyll basis. Since the chlorophyll/protein ratio changes considerably, the normalisation to these key proteins provides a more straightforward and reliable view of the thylakoid proteome between different growth light intensities (Figure 7). Our findings, summarised in the diagram in Figure 7, show both familiar patterns long associated with photosynthetic acclimation such as the decrease of LHCII and the increase in cyt*b*_6_ *f* and ATP synthase levels (Anderson, 1986; Anderson *et al*., 1988; Walters, 2005; Schöttler and Tóth, 2014) with growth irradiance, in addition to previously unreported changes in key regulatory proteins such as PGR6, PGR5, PGRL1B, CURT1, RIQ and STN7/8.

### Features of acclimation to high growth irradiance

The HL plants in this study showed no outward signs of stress and the increased A CO_2_ and ETR(II) values compared to the ML control suggest that they have successfully acclimated to the increased irradiance. The increased LET positively correlated with the increases in ATP synthase and three electron transfer complexes; cyt*b*_6_ *f*, FNR2/TIC62 and PGR6 (Figure 4i). These findings are in line with previous studies that have shown that cyt*b*_6_ *f* plays a major role in LET flux control, with antisense repression of PETC and the use of cyt*b*_6_ *f* inhibitors causing a proportional decrease in LET and CO_2_ assimilation (Anderson *et al*., 1997; Price *et al*., 1998; Kirchhoff *et al*., 2000; Yamori *et al*., 2011). Similarly, minor reductions in FNR levels severely affected LET and CO_2_ assimilation and growth (Hajirezaei *et al*., 2002). Previously it was noted that the concentration of PQ per PSII RC estimated by chlorophyll fluorescence induction with and without DCMU increases with growth irradiance (Gray *et al*., 1996). This increase in PQ per PSII RC is consistent with the increased abundance of the PGR6 protein which has been shown to augment the photoactive PQ pool (Pralon *et al*., 2019). The strategy of the plant is therefore to increase those elements of the LET chain that might otherwise limit flux. Increased levels of PQ and cyt*b*_6_ *f* are in agreement with the more efficient oxidation of Q_A_^−^ in the ML and especially HL plants compared to LL that we observed (Figure 4d). The lower Y(ND) in ML and HL can also be attributed to the increase in cyt*b*_6_ *f* ensuring that electron flux through the LET chain to PSI is more efficient (Figure 4f). Indeed, a similar decrease in Y(ND) and an increase in ETR(II) and A CO_2_ as seen here in HL grown plants have previously been observed in plants overexpressing PETC that accumulate increased levels of cyt*b*_6_ *f* (Simkin *et al*., 2017; Ermakova *et al*., 2019). In many plant species Pc has been positively correlated with photosynthetic capacity (Burkey, 1993; Burkey, 1994; Burkey and Wells, 1996), but in Arabidopsis we observe no such correlation. This finding is consistent with previous reports that PC levels can be reduced by approximately 90% in Arabidopsis by knock‐out of the *PETE2* gene without affecting LET capacity (Pesaresi *et al*., 2009b) and the tendency of this plant to accumulate much higher levels of the protein compared to other species (Schöttler and Tóth, 2014).

A particular problem associated with growth under high irradiance is the potential for the excitation of the RCs by the light‐harvesting antenna system to exceed the capacity of downstream electron sinks. Left unchecked this excess excitation energy and the associated over‐reduction of PSI and PSII acceptor sides has the potential to sensitise the formation of ROS that damage the photosynthetic machinery. Two primary photoprotective mechanisms exist: photosynthetic control and qE, which protect PSI and PSII, respectively. There are strongly contrasting responses of these two mechanisms in HL grown plants; while qE capacity increased (Figure 5f), the amount of photosynthetic control, as shown by Y(ND), decreased compared to ML and LL plants (Figure 4f). Since both mechanisms are induced by build‐up of ΔpH, this seems to be a discrepancy. One possible explanation is that the higher flux through the Calvin–Benson cycle (Figure 4a) and greater abundance of ATP synthase (Figure 2a) means a lower steady state ΔpH is achieved in the HL plants, hence lower Y(ND). In this scenario, the higher levels of qE in HL plants can be explained by the increased relative abundance of VDE, which accelerates zeaxanthin synthesis, and PSBS, both of which have been shown to adjust the ΔpH sensitivity of qE (Ruban, 2016). The strong correlation we observe between PSBS and qE is consistent with studies showing its overexpression increases photoprotective capacity (Li *et al*., 2002).

To our knowledge, changes in CET capacity have not previously been compared with changes in the relative abundance of key CET proteins in plants acclimated to different growth light intensities. Whether CET protects PSI primarily through induction of photosynthetic control Y(ND) or by increasing the amount of ATP relative to NADPH to avoid acceptor side limitation remains unclear, though both factors may be important (Yamamoto and Shikanai, 2019). Here we employed two methods to assess CET: ΔETR (Kou *et al*., 2013) and P700 oxidation (Joliot and Johnson, 2011). The two methods are not in perfect correlation (Figure 5d), which may reflect the fact that they assess CET under quite starkly different conditions, i.e. light activated leaves (Calvin cycle active) and using light absorbed by both PSI and PSII in the ΔETR method and dark‐adapted leaves (Calvin cycle inactive) and light preferentially absorbed by PSI in the P700 oxidation method. Irrespective of the method employed, we observed increasing CET capacity in the order LL < ML < HL (Figure 5b,c). Interestingly, NDH correlated with both CET measures much better than PGR5, despite the latter generally being assumed to facilitate the dominant pathway in Arabidopsis (Yamori and Shikanai, 2016) (Figure 5d). This result is consistent with results in the *hcef1* mutant of Arabidopsis which compensates for increased ATP demand due to a mutation in the Calvin cycle enzyme fructose‐1,6‐bisphosphatase by increasing CET through higher levels of NDH but not PGR5 (Livingston *et al*., 2010). Despite the strong homology between PGRL1A and B, light‐dependent increases were only observed in the latter, suggesting expression of the two proteins may be differentially regulated. Unlike PGR5, PGRL1B did show a strong correlation with CET measured via the ΔΕTR method (Figure 5d). It is interesting in this respect that PGR5 is sub‐stoichiometric with respect to PGRL1, suggesting it may only be needed transiently during the CET process (Hertle *et al*., 2013). Alternatively, if PGR5/PGRL1 serve regulatory roles as suggested by Johnson and Joliot (2011), then the increase in CET capacity could be instead mediated by the higher amounts of FNR and its membrane tethering protein TIC62 (Figure 4h). Membrane tethering of FNR has been suggested to regulate the balance between LET and CET by increasing electron flow from Fd to cyt*b*_6_ *f*, with enhanced amounts of tethered FNR found in C4 bundle sheath cells that perform CET (Goss and Hanke, 2014). In line with this, we found TIC62 and FNR positively correlate with CET (Figure 5d). In addition to an enhanced photoprotective capacity via NPQ, many of the key components of the PSII repair cycle also increased in HL plants. An important factor that may enhance PSII repair in HL plants is the replacement of LHCB4.1 and 4.2, which decrease in HL, with the third isoform of CP29, LHCB4.3, which lacks the large C‐terminal domain present in the former two isoforms. The cryo‐EM structure of the C_2_S_2_M_2_ supercomplex from spinach shows that the C‐terminal domains of LHCB4.1 and 4.2 interact on the stromal side of the complex with CP47, suggesting a role as an anchor (Su *et al*., 2017). Replacement by LHCB4.3 in the supercomplex could remove this interaction, perhaps allowing more straightforward disassembly of the PSII supercomplex to facilitate either PSII repair or NPQ. These changes in PSII structure and PSII repair cycle machinery may act synergistically with the smaller thylakoid grana and reduced stacking seen in HL plants to enhance the speed of PSII repair (Herbstova *et al*., 2012). Previously, the decreased grana size in HL plants has generally been solely attributed to the lower levels of LHCII trimers given their central role in grana formation (Day *et al*., 1984; Anderson *et al*., 1988; Walters, 2005; Schöttler and Tóth, 2014). However, the recent discovery of a crucial role of the CURT1 (Armbruster *et al*., 2013) and RIQ (Yokoyama *et al*., 2016) proteins raises the question of whether they have any role in acclimation induced changes in thylakoid structure. Interestingly, our data show that while LHCII levels are positively correlated with grana diameter (Figure 3f), CURT1A, B and RIQ1 and 2 proteins are negatively correlated with grana stacking. This may reflect the differing roles of these proteins in regulating grana size, with the curvature‐inducing CURT1 protein level affecting the relative areas of the margin domains versus grana core and so number of layers per stack. In contrast, the amount of LHCII that can interact with PSI may be more limited (by availability of binding sites) than PSII and thus increasing LHCII tends to increase grana diameter. In addition to the acclimation‐related changes in grana size described, rapidly reversible changes that are dependent on STN7 LHCII and STN8 PSII phosphorylation have also been observed (reviewed in Johnson and Wientjes, 2020). Accordingly, grana size is reduced when LHCII phosphorylation is at a maximum under low light conditions, whereas it is increased when LHCII is dephosphorylated in both high light and darkness. It is clear that this short‐term response to HL therefore behaves quite oppositely to the long‐term acclimation response seen here, since LL plants have larger grana than HL acclimated plants. This is not surprising since the aim of short‐term high light responses is generally photoprotection through down‐regulation of light harvesting and electron transfer, whereas the long‐term aim is to restore homeostasis by adjusting the sink capacity of the system to better utilise the increased light level. It is interesting that the levels of STN7 and STN8 both increase in HL plants, consistent with the transition to smaller grana under these conditions.

### Features of acclimation to low growth irradiance

Previous studies have highlighted how, in low light, plants generally expand their light harvesting antenna system relative to the PSII reaction centre, but have a generally lower maximum LET capacity (Chow and Anderson, 1987; Chow and Hope, 1987; Chow *et al*., 1988; Bailey *et al*., 2001; Adams *et al*., 2007; Ballottari *et al*., 2007; Petersen *et al*., 2011; Miller *et al*., 2017; Schumann *et al*., 2017). Consistent with these findings, our MS analysis of the light‐harvesting proteins in Arabidopsis showed an increased relative abundance of the major trimeric LHCII complex components LHCB1 and 2 in LL compared to HL (Figure 2a,b), consistent with the chlorophyll *a/b* ratios and biochemical analysis (Figure 1b,d). Since the larger PSII antenna in LL plants confers no benefit in terms of enhanced ETR(II) at low light intensities, the benefit of this acclimation strategy is unclear. Previously, it was suggested that LHCII is considerably cheaper in terms of nitrogen cost to the plant than PSII RCs, which are subject to damage during their normal operation (Evans, 1987). This idea is consistent with the lower light compensation point we observed in LL due to their lower levels of respiration compared to HL plants. Therefore, decreasing the ratio of PSII RCs to LHCII during acclimation to low irradiance allows LL plants to save energy and thereby increase A CO_2_ relative to ML and HL plants. The larger grana observed under low light may allow more chlorophyll to be packed into a given volume of chloroplast, increasing the ratio of light‐dependent reaction components of photosynthesis relative to the Calvin–Benson cycle enzymes and other metabolic machinery, an obvious advantage under light‐limited conditions. A disadvantage of larger grana diameter is the slowing of LET, resulting from increased diffusion distance for the mobile electron carriers PQ and PC (Kirchhoff, 2014; Wood *et al*., 2018); the increase in PC in LL may be necessary to mitigate this effect (Figure 4h).

An unexpectedly increased protein in LL was LCNP, which is required for the photoprotective part of the slowly relaxing qI‐type quenching (Malnoë *et al*., 2018). This suggests that LL grown plants may favour more sustained mechanisms of photoprotection, i.e. qI rather than qE. Another change we observe in LL plants is an increase in the PSI/PSII RC ratio in LL (Figure 2a), in contrast to that seen in pea (Albanese *et al*., 2018) but in line with previous work in Arabidopsis (Bailey *et al*., 2001). We also observed more LHCII associated with PSI and/or in the stromal lamellae fraction under LL (Figure 1d). Since PSII is more efficient at absorbing red and blue light (Wientjes *et al*., 2013a), the increased PSI content, as well as an increased PSI antenna size by augmentation with LHCII (Figure 1d,e) and LHCI (Figure 2c), could reflect an attempt to balance the relative excitation level of the photosystems to optimise LET when light is limiting. An alternative suggestion made by Bailey *et al*., 2001 is that increased PSI levels in LL acclimated plants are required to increase CET to maintain ΔpH at a level sufficient for the generation of ATP when proton deposition by LET into the lumen is low or to make up an ATP shortfall arising from lower respiratory activity. Indeed, it has been shown that the basal leakage of protons from the thylakoid lumen is relatively more important under low irradiance since it increases linearly, whereas ATP synthesis increases exponentially with lumen proton concentration (Berry and Rumberg, 1996). However, we observed no increase in CET capacity of the plants in LL that would support this explanation for increased PSI levels in LL (Figure 5). Furthermore, the abundance of NDH decreases significantly in LL, contrary to the suggestion that this complex plays a crucial role in LL (Yamori *et al*., 2015).

In this study we have provided data showing how nearly 402 thylakoid proteins change in abundance as a result of developmental acclimation to light intensity. Utilising the MS data provided by this study, future work should now attempt to address how the thylakoid proteome responds to natural environments where light, temperature and water availability can be much more variable than the growth room conditions used here (Schumann *et al*., 2017). Previous studies have indicated that plants adopt some of the features of both low and high light plants in such environments, and that the functional photosynthetic properties they show should be modified accordingly (Schumann *et al*., 2017; Vialet‐Chabrand *et al*., 2017). A recent study by Niedermaier *et al*., 2020 found that a fluctuating light environment induced upregulation of proteins involved in photoprotection and CET, while the chlorophyll biosynthesis enzymes decreased in abundance. If this kind of proteomic approach can be widened to include stromal photosynthetic proteins involved in carbon assimilation, combined with transcriptomics and metabolomics and mutants in acclimation (Pesaresi *et al*., 2009a), we can begin to unravel the complex regulatory networks that lead to optimisation of photosynthesis in a changing environment.

## EXPERIMENTAL PROCEDURES

### Growth and acclimation of Arabidopsis

*Arabidopsis thaliana* plants (Col‐0) (15 per light condition) were grown on John Innes M3 compost mixed at a 4:1:1 ratio with perlite and vermiculite, respectively, in a Conviron plant growth room under fluorescent bulbs (emission spectrum shown in Figure S1) at 60% relative humidity, 21°C daytime, 15°C night time temperatures, at a light intensity of 150 μmol photons m^−2^ sec^−1^ with a 12 h photoperiod for 2 weeks until rosettes reached a diameter of around 3 cm. The plants were then transferred to LL (25), ML (150), or HL (800 μmol photons m^−2^ sec^−1^) by moving closer or further from the lighting source. All other conditions remained the same. The cabinet temperature regulation ensured that leaf temperature varied no more than ±2°C under the three light intensities. Plants were acclimated for different lengths of time to account for faster maturation under higher light intensity. HL plants were harvested at 4 weeks, ML plants were harvested at 5 weeks, while LL plants were harvested at 7 weeks, with all plants harvested prior to flowering.

### Chlorophyll fluorescence and P700 absorption spectroscopy

Pulse‐amplitude modulated chlorophyll fluorescence was measured and P700 absorption spectroscopy was carried out using a Dual‐KLAS‐NIR photosynthesis analyser (Walz, Germany) (Klughammer and Schreiber, 2016) on LL, ML or HL plants dark‐adapted for 1 h. Maximum P700 absorption was determined using a 300‐ms saturating pulse of 18 000 μmol photons m^−2^ sec^−1^ in the presence of 255 μmol photons m^−2^ sec^−1^ far‐red light (740 nm). The intensity of far‐red light used was given according to the values provided by Walz Dual‐KLAS NIR software. Chlorophyll fluorescence parameters and the relative P700 redox state were determined at each light intensity using 12 μmol photons m^−2^ sec^−1^ modulated measuring light (540 nm) in combination with a saturating pulse of 18 000 μmol photons m^−2^ sec^−1^. Actinic light was provided at a ratio of 10% 460 nm, 90% 635 nm. Fluorescence parameters were calculated according to Maxwell and Johnson (2000) and P700 parameters according to Klughammer and Schreiber (1994). ETR(I) and ETR(II) values were corrected for leaf absorption using an integrating sphere and for the relative partition of light between PSI and PSII, taken from the ratio of F685 versus F735 77 K fluorescence emission (Figure 1e), taking into account the vibronic satellite contribution of PSII to PSI fluorescence at F735 (Ruban *et al*., 2006). Relative CET capacity was assessed using the method of Joliot and Johnson (2011). Briefly, dark‐adapted plants were given a 200‐ms flash of 650 nm light (2000 μmol photons m^−2^ sec^−1^), then 5 sec of dark before being illuminated with 255 μmol photons m^−2^ sec^−1^ far‐red light (740 nm) to induce PSI oxidation. The half‐time for the rise in P700 oxidation from the moment the far‐red light was switched on was taken as a measure of CET efficiency. Longer half‐times, i.e. delayed P700 oxidation, reflected more efficient CET. CET was also quantified by subtracting ETR(II) from ETR(I) values as described by Kou *et al*., 2013, controls utilised leaves infiltrated with either 1 mm MV or 50 μm AA in 20 mm HEPES pH 7.6 and 150 mm sorbitol.

### Infra‐red gas exchange analysis

CO_2_ assimilation was measured using an LI‐6800 portable photosynthesis system (LiCor, Lincoln, NE, USA) on mature leaves attached to the plants during the middle of the day, that is, from 3 to 6 h into the photoperiod. Relative humidity of the chamber (6 cm × 6 cm) was maintained at 60%, flow rate was 150 μmol sec^−1^ and the block temperature was 20°C. Sample CO_2_ was maintained at 400 ppm and light was held at each intensity for 5 min, a sufficient time to reach steady state. Leaves were acclimated to 25 (LL plants), 150 (ML) or 800 μmol photons m^−2^ sec^−1^ (HL) of light for 20 min prior to the start of each curve (running low to high light intensity) to ensure stomata were open and at their normal aperture size prior to measurement. Light compensation points were calculated individually for each leaf using the method described by Lobo *et al*. (2013).

### Electron microscopy of leaf thin sections

Leaf discs were taken at the point of harvest from positions in the centre of exposed leaves. Electron micrographs of leaf thin sections were obtained according to Wood *et al*.(2018).

### Structured illumination microscopy

Samples were prepared, imaged and analysed according to Wood *et al*. (2019).

### Isolation of thylakoid membranes

Thylakoid membranes were isolated according to Albertsson *et al*. (1994), with the addition of 10 mm NaF to all buffers.

### Chlorophyll analysis

Absorption spectra were taken on an Agilent Technologies Cary 60 UV‐VIS spectrophotometer. Chlorophyll concentration and chlorophyll *a* to *b* ratios were determined according to Porra *et al*. (1989).

### BN‐PAGE

Stromal lamellae were solubilised at 0.5 mg ml^−1^ Chl in 2% digitonin, 50 mm Bis‐Tris pH 7.2, 10 mm NaF and 10% glycerol, for 1 h on ice. Grana membranes were solubilised in 0.5% *n*‐hexadecyl β‐d‐maltoside, 0.2% *n*‐dodecyl α‐d‐maltoside, 50 mm Bis‐Tris pH 7.2, 10 mm NaF and 10% glycerol, for 1 h on ice. Solubilised protein complexes were isolated and separated by BN‐PAGE, as previously described (Wood *et al*., 2019), before Coomassie staining and imaging.

### Low‐temperature fluorescence spectroscopy

The 77 K fluorescence spectroscopy was carried out as previously described (Wood *et al*., 2019).

### Thylakoid membrane protein extraction and proteolytic digestion

Thylakoid membranes were solubilised by sonication in 1% (w/v) SL as described previously (Lin *et al*., 2013). Starch granules were then removed by centrifugation at 10 000 ***g*** for 2 min. Aliquots of the supernatant containing 50 µg protein (Bio‐Rad DC assay) were adjusted to 15 µl with 1% (w/v) SL, 100 mm triethylammonium bicarbonate (TEAB) pH 8.5 and then reduced by the addition of 1.5 µl 100 mm tris(2‐carboxyethyl) phosphine‐HCl and incubation at 37°C for 30 min. Proteins were S‐alkylated by the addition of 1.5 µl of 200 mm iodoacetamide in 100 mm TEAB pH 8.5 and incubation at ambient temperature in the dark for 30 min. Samples were adjusted to 50 µl with 1% (w/v) SL and 100 mm TEAB pH 8.5 and proteolytic digestion was carried out after the addition of 2 µg pre‐mixed trypsin/endoproteinase Lys‐C (Promega, Madison, WI, USA) for 3 h at 37°C. Extraction of SL was performed as previously described (Lin *et al*., 2013) by adding an equal volume of ethyl acetate and acidification with 10 µl 10% (v/v) trifluoroacetic acid (TFA). The samples were vortexed for 1 min and then centrifuged at 15 700** ***g*** for 5 min to accelerate phase separation. The peptide‐containing lower phase was isolated, dried by vacuum centrifugation and dissolved in 50 µl 0.5% (v/v) TFA and 3% (v/v) acetonitrile before desalting with C18 spin columns (Thermo Scientific, Waltham, MA, USA) according to the manufacturer’s protocol. The peptides were again dried by vacuum centrifugation and stored at −20°C.

### Analysis by mass spectrometry and protein identification

For analysis by nano‐LC‐MS/MS, the peptides were dissolved in 0.5% (v/v) TFA and 3% (v/v) acetonitrile, and 400 ng of each of three biological replicates was analysed in triplicate in randomised order. Peptides were resolved on an EASY‐Spray PepMap RSLC C_18_ column (Thermo Scientific, 50 cm × 75 μm ID, 2 μm, 40°C) with the following gradient profile delivered at 300 nl min^−1^ by a Dionex RSLCnano chromatography system (Thermo Scientific): 97% solvent A (0.1% formic acid in water) to 10% solvent B (0.08% formic acid in 80% acetonitrile) over 5 min, then 10% to 50% solvent B over 3 h. The mass spectrometer was a Q Exactive HF hybrid quadrupole‐Orbitrap system (Thermo Scientific) programmed for data‐dependent acquisition with profile full MS scans at 120 000 resolution and a maximum of ten centroid product ion scans at 30 000 resolution per cycle. Proteins were identified by searching the MS data files against the *A. thaliana* reference proteome database (www.uniprot.org/proteomes/UP000006548, downloaded on 10 December 2018) using MaxQuant v. 1.6.3.4 (Cox and Mann, 2008) with the intensity‐based absolute quantification (iBAQ) (Cox and Mann, 2008; Schwanhäusser *et al*., 2011) option selected. Search parameters were: carbamidomethyl‐Cys (fixed modification), Met oxidation, protein N‐terminal acetylation, Lys acetylation and Gln to pyro‐Glu conversion (variable modifications) with a maximum of two missed cleavages.

### Mass spectrometry‐based protein quantification

Quantification results in the form of iBAQ (Cox and Mann, 2008; Schwanhäusser *et al*., 2011) intensities, as generated by MaxQuant (Cox and Mann, 2008) for the identified proteins, were processed using Perseus v. 1.6.2.3 (Tyanova *et al*., 2016). To compensate for variation due to sample loading and MS spectral acquisition timing, iBAQ intensities for the target proteins were normalised to the intra‐analysis sum of iBAQ intensities of key photosynthetic complexes PSII (PSBA, PSBB, PSBC, PSBD, PSBE, PSBF, PSBH, PSBO1, PSBO2, PSBP1, PSBP2, PSBQ1, PSBQ2, PSBR), PSI (PSAA, PSAB, PSAC, PSAD, PSAE1, PSAE2, PSAF, PSAG, PSAH, PSAK, PSAL, PSAN, PSAO), cyt*b*_6_ *f* (PETA, PETB, PETC, PETD) and ATP synthase (ATPA, ATPB, ATPC, ATPD, ATPE, ATPF, ATPH, ATPI). Normalised iBAQ intensities for each MS analysis are provided in Table S2. The significance of changes in protein expression following acclimation to low, moderate and high light growth conditions was determined using a modified Welch *t*‐test as implemented in Perseus (Tyanova *et al*., 2016). Protein identifications were assigned as being associated with the thylakoid membrane, lumen or plastoglobules using SUBA4 (Hooper *et al*., 2017).

### SDS‐PAGE and immunoblotting

Thylakoid membranes were solubilised in NuPAGE LDS sample buffer for 1 h at ambient temperature and then separated by SDS‐PAGE on Invitrogen 12% Bis‐Tris NuPAGE gels (Thermo Fisher Scientific). SDS‐PAGE sample loading was normalised to equal amounts of chlorophyll. Immunoblotting was performed as described by Proctor *et al*. (2018), with antibodies raised against PSBD, PSBA, PETA and ATPH (Agrisera, Vännäs, Sweden).

## AUTHOR CONTRIBUTIONS

SEF, MJD, CNH, PJJ and MPJ designed the experiments. SEF, PJJ, CH, FP and WHJ performed the experiments. PJJ, MJD, SEF, CNH and MPJ wrote the manuscript. All authors proof‐read and approved the manuscript.

## CONFLICT OF INTEREST

The authors have no conflict of interest to declare.

## Supporting information


**Figure S1.** Growth light emission spectrum. Emission spectrum of fluorescent lighting used for Arabidopsis growth.

**Figure S2.** P700^+^ oxidation kinetics. Dark‐adapted plants were given a 200‐ms flash of 650 nm light (2000 μmol photons m^−2^ sec^−1^), then 5 sec of dark before being illuminated with 255 μmol photons m^−2^ sec^−1^ far‐red light (740 nm) to induce PSI oxidation. *N* = 4 for each curve.

**Table S1.** Relative abundance of thylakoid proteins calculated using label‐free quantitative proteomics. Details of the 402 quantified thylakoid proteins including functional category, protein name, description, UniProtKB identifier, AGI code and abundance ratios. Proteins with altered abundance in different light irradiances were identified by a modified one‐way anova (*q* < 0.05). Significant proteins were subjected to further statistical analysis to determine significant changes between light conditions by a modified Welch *t*‐test (*q* < 0.05). Statistical tests (anova and Welch’s *t*‐test) were implemented in Perseus (Tyanova *et al*., 2016) with a 5% permutation‐based false discovery rate calculated from 250 randomizations. Median protein iBAQ (Cox and Mann, 2008; Schwanhäusser *et al*., 2011) values for each light condition were used for the calculation of ratios in ML versus LL, HL versus ML and HL versus LL.

**Table S2.** Intensity‐based absolute quantification (iBAQ) of Arabidopsis thylakoid proteins. MS‐identified protein iBAQ (Cox and Mann, 2008; Schwanhäusser *et al*., 2011) values following normalisation to the intra‐analysis sum of key photosynthetic complexes PSII (PSBA, PSBB, PSBC, PSBD, PSBE, PSBF, PSBH, PSBO1, PSBO2, PSBP1, PSBP2, PSBQ1, PSBQ2, PSBR), PSI (PSAA, PSAB, PSAC, PSAD, PSAE1, PSAE2, PSAF, PSAG, PSAH, PSAK, PSAL, PSAN, PSAO), cyt*b*_6_ *f* (PETA, PETB, PETC, PETD) and ATP synthase (ATPA, ATPB, ATPC, ATPD, ATPE, ATPF, ATPH, ATPI) with supporting information generated from MaxQuant (Cox and Mann, 2008): number of peptides identified, protein sequence coverage, identification score (derived from peptide posterior error probabilities) and MS/MS count. Identifying information from the *Arabidopsis thaliana* UniProtKB proteome database is given as majority protein IDs, protein names and gene names.

## Data Availability

The mass spectrometry proteomics data have been deposited to the ProteomeXchange Consortium via the PRIDE partner repository (http://proteomecentral.proteomexchange.org) with the data set identifier PXD014982. All other data can be obtained from the corresponding authors upon request. The following figures have associated raw data: Figures 2–7. Quantitative MS analysis results are provided in Table S1.
