# Supplementary figures and images for: Developmental acclimation of the thylakoid proteome to light intensity in Arabidopsis
Source: Plant J. 2020 Nov 27;105(1):223–44. doi: 10.1111/tpj.15053 (PMC7898487; doi:10.1111/tpj.15053)

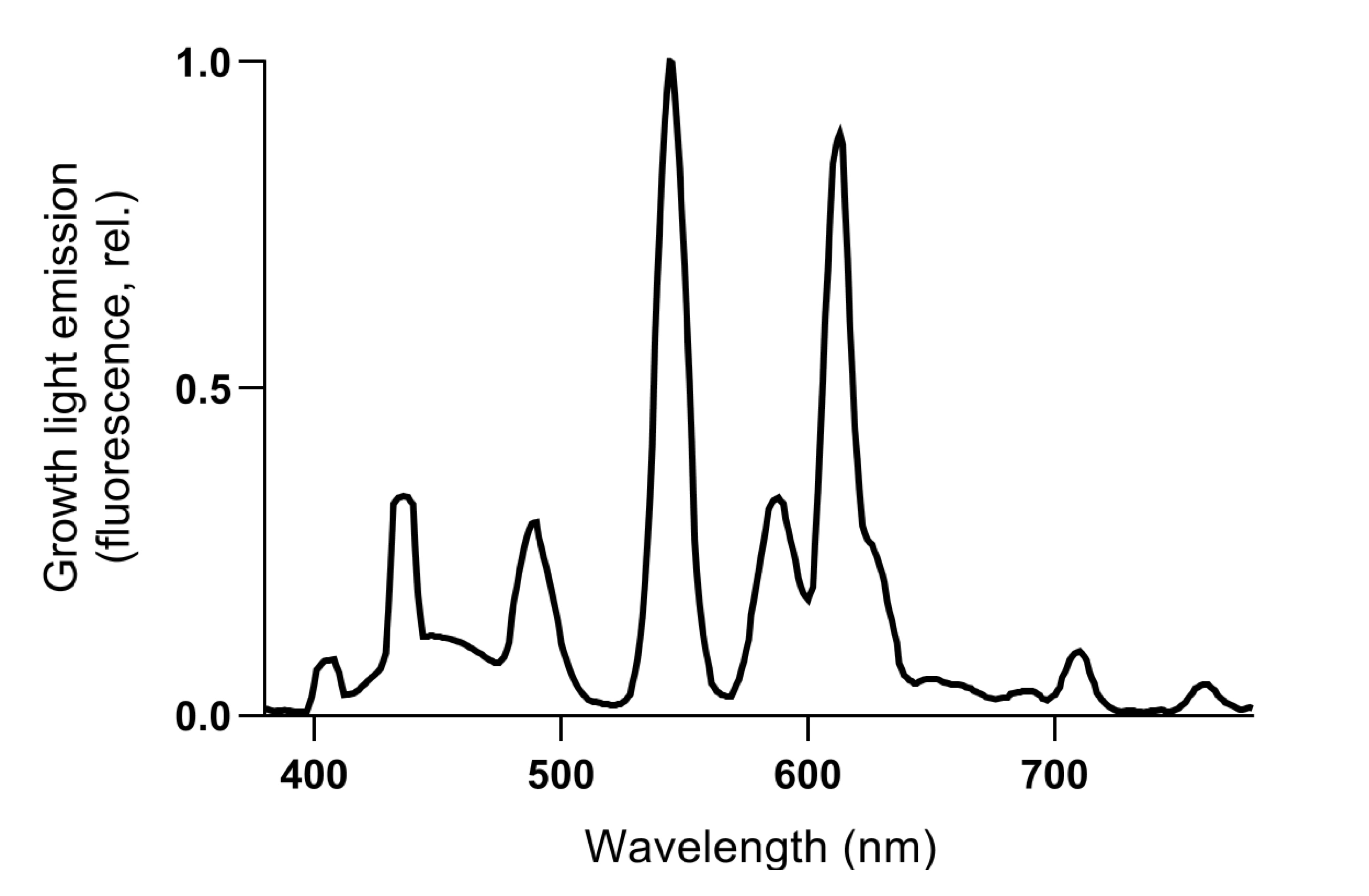

Supplement: Supplementary file 1 — Figure S1. Growth light emission spectrum. Emission spectrum of fluorescent lighting used for Arabidopsis growth. [file TPJ-105-223-s001.jpg]

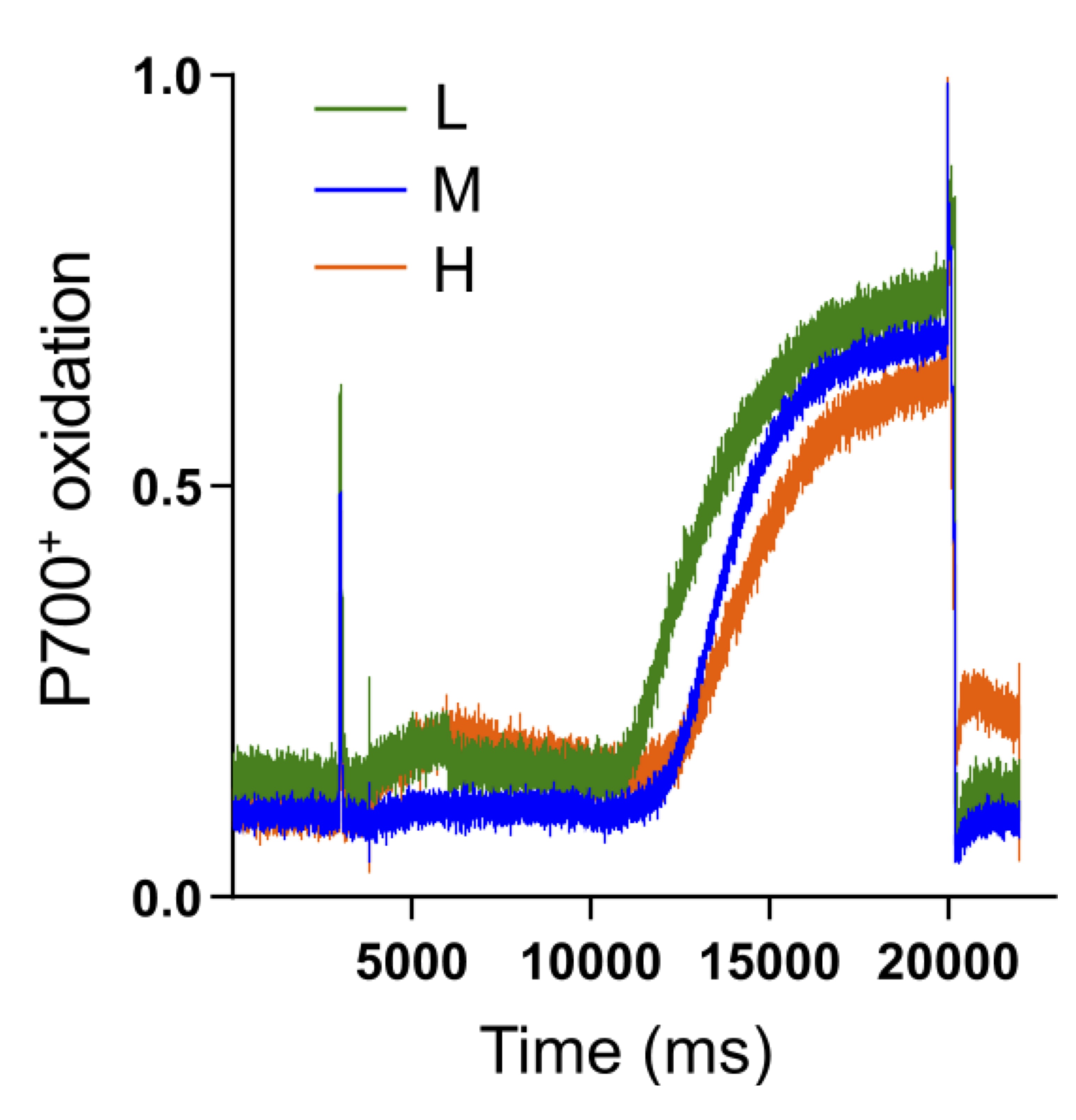

Supplement: Supplementary file 2 — Figure S2. P700+ oxidation kinetics. Dark‐adapted plants were given a 200‐ms flash of 650 nm light (2000 μmol photons m−2 sec−1), then 5 sec of dark before being illuminated with 255 μmol photons m−2 sec−1 far‐red light (740 nm) to induce PSI oxidation. N = 4 for each curve. [file TPJ-105-223-s002.jpg]
